# Data Work: Meaning-Making in the Era of Data-Rich Medicine

**DOI:** 10.2196/11672

**Published:** 2019-07-09

**Authors:** Amelia Fiske, Barbara Prainsack, Alena Buyx

**Affiliations:** 1 Institute for History and Ethics of Medicine Technical University of Munich School of Medicine Technical University of Munich Munich Germany; 2 Department of Anthropology University of North Carolina at Chapel Hill Chapel Hill, NC United States; 3 Department of Political Science University of Vienna Vienna Austria; 4 Department of Global Health & Social Medicine King's College London London United Kingdom

**Keywords:** big data, data work, medical informatics, internet, data interpretation, decision support systems

## Abstract

In the era of data-rich medicine, an increasing number of domains of people’s lives are *datafied* and rendered usable for health care purposes. Yet, deriving insights for clinical practice and individual life choices and deciding what data or information should be used for this purpose pose difficult challenges that require tremendous time, resources, and skill. Thus, big data not only promises new clinical insights but also generates new—and heretofore largely unarticulated—forms of work for patients, families, and health care providers alike. Building on science studies, medical informatics, Anselm Strauss and colleagues’ concept of patient work, and subsequent elaborations of articulation work, in this article, we analyze the forms of work engendered by the need to make data and information *actionable* for the treatment decisions and lives of individual patients. We outline three areas of data work, which we characterize as the work of supporting digital data practices, the work of interpretation and contextualization, and the work of inclusion and interaction. This is a first step toward naming and making visible these forms of work in order that they can be adequately seen, rewarded, and assessed in the future. We argue that making data work visible is also necessary to ensure that the insights of big and diverse datasets can be applied in meaningful and equitable ways for better health care.

## Introducing Data Work

With health care becoming increasingly data driven, more and more domains of people’s lives are *datafied*, that is, they are translated into a format that lends itself to automatic processing and computation. Examples range from data generated by individuals using health and lifestyle smartphone apps, the digitalization of health records, data from direct-to-consumer testing or drug trials, to biobanking research and clinical genetic testing. Data from increasingly diverse sources are thus rendered, at least in principle, usable for health care purposes. Yet, deriving insights for clinical practice and individual life choices, and deciding what data or information should be used for these purposes, poses difficult challenges. Indeed, it has been argued that “big data won’t cure us” [1]; turning data into meaningful information for clinical practice requires tremendous time, resources, and skill. Thus, big data not only promises new clinical insights but also generates new—and largely unarticulated—forms of work for patients, families, and health care providers alike.

Building on insights from science studies, medical informatics, as well as on the concept of patient work and subsequent elaborations of articulation work [2-4], in this article, we analyze the forms of work engendered by the need to make data and information *actionable* in the health care context [5]. Doing so brings the perspective of social and ethical studies of biomedicine into conversations around digital medicine, emerging technologies, medical devices, apps, engineering, and informatics. We outline 3 areas of data work, which we characterize as the work of (1) supporting digital data practices; (2) interpretation and contextualization; and (3) inclusion and interaction. We argue that it is necessary to name and make visible these forms of data work for them to be adequately acknowledged, assessed, and rewarded. Making data work visible can also help to ensure that the insights of big and diverse datasets can be applied in meaningful and equitable ways for better health care. Although this paper primarily aims to highlight emerging forms of work in the era of data-rich medicine that have not been explicitly or comprehensively considered heretofore, we close by outlining avenues for future practice and policy.

## Data Work: A Persistent Challenge for the Era of Data-Rich Medicine

### Emerging Forms of Data Work

Controversies surrounding data use, storage, and sharing illustrate the important ethical questions that emerge when data collection and analyses are applied to new ends. Examples in the news abound, for instance, the rise of direct-to-consumer genetic testing for diseases such as cancer, seen recently through the example given on National Public Radio of an uninsured American woman concerned about her risk of breast cancer [6]. Upon reading her results from 23andMe, the woman admitted feeling less urgency about getting additional testing or mammograms with her physician—something that geneticists worry could pose problems for individuals carrying variants undetected by tests offered by commercial sources, or for those who receive summary advice from individuals without proper training, possibly leading to clinical harm in the future. Other disputes have emerged when health technologies are applied to new ends, such as the recent identification of the *Golden State Killer* in California, United States, in April 2018. Detectives were able to identify the perpetrator by matching crime scene evidence with a family member’s DNA profile that the family member had uploaded to a genealogy website. The incident, and subsequent admission that private companies have shared access to their database with law enforcement to find potential suspects, spurred controversy among experts and the public over the legitimacy of using the personal data of volunteers who had not consented to such law enforcement applications [7,8]. Controversies such as these—as well as others surrounding privacy and, for example, the hacking of medical devices [9], or matters of justice and fairness in algorithms [10]—point to the centrality of data at the heart of negotiations over the public good; the status of data generated outside of official forums of science and medicine; and central ethical questions of privacy, consent, and benefit that are emerging in new configurations [11,12].

By *data work*, we are referring broadly to the forms of technological, analytical, and emotional work undertaken by all actors within the health care system that is necessary to make data clinically and personally meaningful. Here, we focus on the emerging forms of data work undertaken by patients and health professionals. This work is already occurring, for example in the interpretation of direct-to-consumer genetic tests [13], efforts to improve patient understanding of broad consent in biobanking [14], or as researchers define proteomic markers of risk, such as for ovarian cancer [15], albeit in an often unrecognized and patchwork manner. Although science studies scholarship has explored various determinants and conditions of data production in the health sphere [16-18], the types of work that are necessary to make diverse forms of health data actionable in daily life by patients and health professionals have not been systematically addressed or conceptually analyzed [19]. *Data work* is ongoing and constitutes a formidable yet underresearched challenge in the era of data-rich medicine. But what kinds of work does this entail, and for whom? What divisions of work or tools would be necessary for addressing ethical and equitable applications of data in everyday life?

Empirical studies examining the organization and structure of medical work from a sociological perspective [20-24] have been helpful to draw attention to the often invisible contributions that patients and their family members make to all aspects of health care. However, conceptualizations of such *patient work* in the era of data-driven medicine are, as of yet, largely missing [25]. As debate grows in medicine over how to best actualize voluminous and diverse data for better outcomes in health care [26,27], many of the biggest challenges are of a social, rather than technical, nature [1]. In this context, more systematic attention to the ways in which professional and nonprofessional actors within the health care system help, for example, to create and interpret data, would fill an important gap. In the following section, we outline and describe three areas of emerging forms of work that have accompanied the turn toward big data in medicine, identify who does this work, and sketch potential ways of addressing concerns that arise in connection with this work. For each area of data work, we offer one vignette to illustrate the forms of data work that are already ongoing. Although the boundaries between these different types of data work are fluid, we posit that there is analytic value in drawing out the key features that characterize each activity to see what challenges they pose and how we might address these.

### More Than a Click Away: Supporting Digital Data Practices

G is excited about a new app that promises to keep track of his heartbeat, steps taken, and minutes slept, and to aggregate these data with his weight, blood pressure, and glucose levels. Yet, after looking at the Terms of Service, he realizes that by using the app he signs the rights to his data over to the company. G wonders if there is another option. Finding himself mired in pages of legalese, he starts to think, “maybe I’m just too uptight—what could they really do with all this data, anyways?”

Advances in mobile devices have changed how health information and support services are being accessed, communicated, monitored, and acted upon [28], offering potential gains ranging from clinical oncology [29] to improving health outcomes for low-income populations [30]. As a result, patients create and engage with health data not only in medical institutions but also in their homes and in other places outside the clinic, via wearable or portable devices, or other tools. Patients and health care professionals alike are faced with ever wider types and larger volumes of data that could *potentially* be relevant for health care, without a clear understanding of the implications of specific forms of personal data [31]. In the domain of mobile apps, one form of emergent data work is the work done by patients who search through the fine print of Terms of Services of new devices and apps to decide whether or not to use them. This is often not easy to do; for instance, the interests of a company providing a digital health device or platform might be hard to fathom for a user, posing potential concerns for individuals who are consenting to data use agreements for a health app, or uploading their medical history to a Web portal for a rare-disease patient community.

Furthermore, the ability to learn about genetic traits—which can now be done with ever lower expense on the internet —raises profound ethical challenges. As Kung and Wu ask, “if we discover certain genetic risk factors in our genome sequences, do we (or our health care providers) have a responsibility to inform our family members who might have similar genetic risks?” [32]. Privacy matters, and the effects of new health technologies on future generations all become important concerns with which individuals have to grapple, while very little or no guidance may be available. The work that people are doing when navigating the landscape of available offers, and in deciding what test they should take and what behavior they should track in an attempt to maintain or increase their health, should not be trivialized. There are increasing expectations that individuals make informed decisions as responsible managers of their health, and now also as owners (morally or legally) of their data.

In addition to such new data work for patients, another novel form of data work emerges for health professionals. This consists of assisting patients and their families in navigating the landscapes of available offers for tests, devices, and services, and helping them to decide whether they should *datafy* certain aspects of their lives and bodies in the first place. This data work includes engaging patients in conversations about the implications of their potential data contributions before patients have had practical experience with these digital practices, and about whether and how they should consider engaging in certain activities. Steering patients through the multitude of options is an important yet complex task. Recent studies have also shown that socioeconomic status, age, English literacy, and digital literacy all play important roles in the uptake of new mobile technologies such as health apps [28,29,33] in engaging in Web-based participatory medical research [34] and in efforts to counter the *digital divide* [35-37]. Importantly, these differences also influence whose data are missing from the broader evidence base upon which future decisions in medicine might be made [25]. This points to the growing need to ensure that such digital health practices and technologies do not exacerbate existing inequalities in society or health and the critical role that health professionals are called upon to play in mediating digital engagements.

Looking forward, we thus anticipate that the data work of professionals in this space will include not only assisting patients in navigating this digitalized network of health-relevant services but also assisting those who cannot, or choose not to, engage digitally [38]. As noted, people who do not make use of digital tools to collect, view, and share data and information about themselves can become *missing bodies* in today’s health care environments, meaning that their bodies, needs, and behaviors remain unaccounted for in decisions made on the basis of new digital health sources [25,39]. Especially when the stakes are so high, neither offering guidance on patient use of digital tools and new health products nor understanding the advantages and disadvantages of the many new products on the market every day is intuitive. To be effective, these activities require time and appropriate training, which are in very short supply in today’s time-starved health care environment [40-43].

One possibility, as we have argued elsewhere, to better support both patients and providers in the era of data-rich medicine would be the creation of a new, intermediary profession entirely, which we have termed *health information counselors* (HICs) [44]. With a broad knowledge of various kinds of health data and data quality evaluation techniques, as well as analytic skills in statistics and data interpretation, our vision is that HICs would be trained also in interpersonal communication, health management, insurance systems, and medico-legal aspects of data privacy. Operating as a clinical consultancy, HICs would have the ability to translate the complex language of data into intelligible and actionable information for both patients and physicians. The creation and implementation of such a specialty would enable patients to make educated, truly autonomous choices about how these novel forms of health data can inform their personal care decisions. Although certainly not the only option for addressing the aforementioned concerns, the creation of this new specialty would go a long way in assisting individuals such as G from our opening vignette, as well as health care professionals, to consider their options and make more informed choices about how increasing amounts of health data and information can or should inform health care.

### How to Tell It All Apart: The Work of Interpretation and Contextualization

A brother informs his sister, L, that he has done a commercial DNA test that revealed that he could be a carrier for a particular condition. Because L is considering having a child with her partner, she wonders if she should undergo testing, and what this would mean for their decisions going forward. In reading the leaflet provided by a company offering the testing, she is not sure what is meant by the information that carrier reports may vary in detection accuracy by ethnicity (L has Ashkenazi heritage), and that carrier testing does not include all possible variants for a given condition. L wonders: “What would this information mean for me personally? Who could I ask about this?” She is unsure if her primary care physician is the right person to ask, and who else she could turn to.

Testing practices such as the one described in this vignette have become a means through which individuals understand themselves and their relationship to society. For some patients, the quantified self can allow people to see new patterns or make changes in their lives: counting steps might lead one to take the stairs, and tracking sleep patterns might lead another to try and get an extra hour of sleep. For others, finding out the percentages of one’s global ancestry or likelihood that they could be a carrier for a genetic condition represents personally significant information. Yet, the effects of health-related data and information are often difficult to anticipate and understand. Randomized controlled trials have studied the clinical impact of patients’ use of mobile and digital health tools, such as the effectiveness of smartphone apps for weight loss and self-applied therapies [45-47]. Other studies have shown the necessity of looking at patient experience of digital tools to understand how mobile health affects self-management of chronic conditions or changes in well-being [48-50]. In some cases, certain forms of health information may have personal utility for some people even if they lack clinical utility [51]. Overall, such research shows that further work—such as prescreening or offering hands-on assistance and consultation—is needed to turn a health app or Web-based service such as direct-to-consumer testing into a meaningful tool for an individual patient [52].

Data science holds the potential to offer important predictive and diagnostic information that can be used to improve decisions taken by clinicians to reduce error or support estimates, such as the likelihood of medication adherence or organ rejection [53,54]. Yet, from body temperature to steps taken, heartbeats, and hydration levels, it is not yet clear what the biometric data collected via devices such as wearables or smartphones will mean for medical practice and health practitioners. The same is true for nonmedical grade testing services. Both the quality of the data and the possibilities of data interpretation are relevant here. Commercial devices are often not calibrated to the standards of medical grade devices, particularly if not used exactly as intended, which means that data collected through them cannot be used as reliable evidence for health care decisions. Internet communities and apps that offer peer-to-peer support can also be problematic when inaccurate or purely anecdotal information is shared, for example, how-to-hack Web-based tutorials or the increasing use of YouTube as a platform for disseminating misleading health information or offering problematic interpretations of existing data on conditions such as anorexia and bulimia [55-58].

The complex task of discerning irrelevant, unreliable, or misleading health information from relevant, valid, and clinically actionable personalized health resources and then interpreting and contextualizing these for specific patients and their families is emerging as a significant, and time-consuming, activity for health care providers. In our survey of health professionals working in the region of Schleswig-Holstein, Germany, providers expressed repeated concerns about the increasing amount of time devoted in patient encounters to explaining why data from a Web-based genetic test are not relevant, or why a novel therapy reported on a patient community website is not the best choice for a family member [59]. These findings are echoed by recent reports that have pointed to the need for new and improved decision aids to situate the most personally relevant and high-quality digital tools for patients [28,60]. Although some standardization work regarding this issue is currently undertaken by groups such as the Consumer Technology Association, the creation of new devices, apps, and programs and the demands these pose regarding data interpretations and contextualization continues to exceed regulatory processes and physician workloads.

In this context, data work includes deciding which data or information are reliable and relevant for a given context of a specific patient—including contexts outside of the clinic—to decide which intervention, tool, or device might be appropriate or helpful in a given situation, or in future. Again, this is a complex task. For example, discerning whether data brought in by patients derived from commercial or hacked devices can be clinically relevant involves researching devices, analyzing the information they collect, and deciding if, and how, the information generated could be used to inform individual case decisions. In some instances, such data work could include contacting the company producing the device for more information, or seeking out additional resources to evaluate the reliability of the data generated. The same is true for commercially available genetic testing, or the results derived from nonstandard forms of research occurring on patient platforms, such as in some citizen science initiatives [61].

The work of contextualization also increasingly extends to the analysis of the algorithms used to produce data in the health care context. Algorithms are neither ‘objective’ nor intrinsically neutral and they can exacerbate societal inequities. Biases—regarding race, gender, educational status, body mass index, and so on—are programmed into systems, and the characteristics of datasets that these systems use to learn might reproduce inequities [10,62]. As more and more parts of our lives are being *datafied*, there is an increasing need for contextualization of the health data gained through Web-based tests, mobile, and digital technologies [63]. This includes making the context of data explicit, and asking questions such as: What data was collected, from whom, and how? What do these data represent, and what do these leave out? How has it been made legible for computation, and what has been lost or gained in the process? Such questions are increasingly necessary given the growing ubiquity of domains of everyday life being understood through computational practices. All of the above forms of evaluation require a significant degree of analytical and computational literacy and reflection on whether a particular process of meaning-making relies on evidence that is accurate and reliable in a technical sense, if it is mostly personal and social, or if it is indeed faulty or misleading [64].

Patients, in addition to health care professionals, are also increasingly participating in specific forms of work, including outside of clinical settings. This is the case, for example, when patients do internet searches and seek assistance in making sense of reports or articles found on the internet, thus engaging in the work of sorting, interpreting, and analyzing diverse and often competing sources of information. Often this type of work is undertaken by family members or caregivers to support a patient’s health care choices. The work of contextualization will remain a persistent challenge in years to come as more devices, apps, health-related services are offered to individuals outside the supervision of medical professionals. As an area that is in need of robust investigation and public debate, it would be productive to have greater involvement by scientific and academic societies in conducting and sharing analysis of how data can and should be used. Although some of this work is already ongoing, such as recent reports addressing the opportunities, risks, and ethical questions associated with use of *good* artificial intelligence (AI) in health care, or developing specific suggestions that can be taken up by stakeholders and policy makers at national and international levels [65,66], further work is needed on different aspects of the use of big data in medicine. By fostering greater debate, and providing material that is available for lay readership to engage with the stakes of their data engagement, academic scholarship can better support digital literacy in this area.

### Facilitating Conversations About Aims and Interests: The Work of Inclusion and Interaction

Upon entering the hospital for an inpatient stay, P, an elderly patient, is asked to opt-in to the institutions’ efforts to improve efficiency and calculate predictive health and frailty scores for patients [67]. P is not sure what this means, or how his personal information will be stored and used in the future. [67]

The prior areas of data work that we have outlined have emphasized the need for a strong awareness of what new data, tests, and technologies are available and how they work. Data-rich medicine highlights a number of ethical issues [11], not least of which is the cross-cutting work of addressing different aims, goals, and interests. As data are increasingly accessible, distributed, revealing, and reidentifiable, ethical concerns pertaining to digital health, large datasets, and precision medicine are multiplying, including issues of consent, protecting participant privacy concerns, and maintaining public trust [68]. Given that many of data-driven practices track new territory in health, questions of power asymmetries and social-economic value are emerging with new relevance [12,69]. An important form of data work thus involves fostering conversations with and across stakeholder groups around these concerns.

As precision medicine moves away from *one size fits all* approaches to treatment, machine learning approaches are increasingly improving the ability to target patients for specific treatments, such as in the use of DNA methylation to subclassify tumors of the central nervous system [70]. The potential of this work to improve personalized therapies through the use of mathematical models is great, yet both the perceived benefits and the social, economic, and health-related concerns vary by actor [71]. In other words, a provider will likely have a different set of investments in the technology, research, and treatment outcomes than a given patient, a hospital chief executive officer, a pharmaceutical company, or an interested member of the public. A patient might be most concerned about loss of privacy, discrimination, or stigmatization (albeit also interested in disease prevention and better treatment), whereas company representatives might be uneasy about losing exclusive access to datasets and find themselves at odds with community members committed to principles of open access. Thus, a central aspect of data work is creating the spaces for interaction and facilitating conversations between differently motivated parties, such as assisting one actor to understand the concerns of another, or finding novel ways to address specific concerns around discrimination, privacy, or equity.

In the digital era, privacy concerns take on a different configuration than in the paper age [72]. Data work in the context of privacy is not limited to simply informing patients of what happens with their data and information once it has been collected but includes moving beyond the widely accepted ethical principle of respecting patient autonomy [73] to including patients in decisions over what type of information will be collected about them in the first place, and to what end. The General Data Protection Regulation (GDPR) introduces protections that began in 2018 across the European Union (EU; including the United Kingdom), but outside of the EU, there is little agreement on regulatory standards for digital health tools or data protection in research, databanks, and big data [61,74-77]. Despite the overall objective of European harmonization, the GDPR gives member states leeway, for instance, in determining whether patient consent is required for secondary data use in medical research, and in which form [74,78]. These national differences have various practical and normative consequences, most of which have not yet been fully analyzed, as well as different implications for research practice across member states. Legislation in countries where data protection is sector specific, rather than general, such as Health Insurance Portability and Accountability Act (HIPAA) in the United States, has addressed data privacy and security concerns relating to medical information since 1996. Subsequently, the HIPAA omnibus rule of 2013 modified the Act to meet guidelines set by the Health Information Technology for Economic and Clinical Health in 2009. Such efforts have expanded the extent of HIPAA beyond providers and insurance companies to also consider the role of business associates. However, even though concerns surrounding patient privacy and the reuse of health information have long been an important topic, the ability of existing regulation such as the GDPR or HIPAA to fully address the concerns emerging in the age of big data remains unknown [79]. We highlight here that the forms of data work we identify can pose particular challenges for privacy, including: the rapid rate of digital innovation; that decisions need to be made on both on the individual and societal level about which aspects of everyday life should be captured by data in the first place; that harm can occur from data use that is not necessarily illegal [80]; as well as broader concerns about data privacy protection legislation.

How to effectively engage a range of stakeholders, including patients, providers, researchers, and insurance companies in these data work concerns, is an ongoing discussion in both clinical practice and biomedical research [81-83]. One critical area of data work for health care providers and researchers is holding conversations with patients about data collection and privacy to better understand the impact of collecting anonymized patient health data in research [14,84]. Data work includes ensuring that patients are party to the decisions about what information will be included in their records, who the gatekeepers for this information are, and for which goals and for whose benefit this information will be used beyond the realm of individual-level health care decisions. It is critical that these discussions include reflections on how data could potentially be reused in the future, for example, the use of predictive health and frailty scores by insurance companies as mentioned in the vignette, as well as the identification of potential protections to guard against uses of data that could be harmful or exclusionary to patients. Specific conditions of access, reuse, and reidentification need to be identified and continually updated in light of new digital advances.

In particular, digital technologies raise important questions over the access of personal information. Each patient’s needs and interests are influenced by their human, natural, and artifactual environments. An individual’s decision to access his or her electronic health records or use a Web-based genetic testing service is not just a choice made by an atomistic individual but an act shaped by the person’s family ties and social relations, his or her connection to others, and the country in which he or she lives [85]. For example, an individual may want to share and discuss this health information with his or her partner or children [82]. This decision to share and discuss information received is not an afterthought but may well have shaped the decision to obtain information in the first place [86]. This layer of dyadic or multilateral forms of decision making can vary significantly across cultural contexts.

In sum, joining distinct datasets from different types, locations, and ethical standards adds additional layers of deliberation to well-rehearsed ethical considerations. Recognition and fostering dialog around aims and goals and the more complex, potentially shared nature of decision making in the era of big data is a critical form of data work. However, how this can be achieved when data are held in dispersed locations and are diverse in nature is entirely unclear. It will require close communication between the patient and the health care provider to ensure that the built-in decisional pathways offered by data-driven practices do not eclipse individual priorities. One potential way of addressing this concern is to reconsider existing methods for ensuring patient privacy and protection and addressing them through regulatory measures, for example through the GDPR in Europe. According to the GDPR, for personal data to be processed lawfully, either individual consent is required, or a legal authorization has to apply. The most relevant legal authorization in the medical context is the research exemption (Article 89). However, particularly in view of international research collaborations, further work is necessary on how GDPR is implemented across individual countries. To provide an example, in line with Article 89, Germany now allows data processing of pseudonymized data for scientific or historical research purposes or for statistical purposes, at least prima facie, without requiring individual consent. However, neither clear guidance exists as of yet for how these purposes are exactly delineated nor have studies been conducted on how this new legal provision has penetrated research practice and how effects differ from countries that are more restrictive. Countries that have long-term experience with more permissive approaches, such as broad or blanket consent (eg, the United Kingdom) and the processing of genetic data should help to anticipate the implications of the novel practice and to raise the standards for how informed consent can be better operationalized in light of the concerns of big data—also in areas outside of Europe [87].

## Who Does Data Work: Patient Work 2.0?

The different kinds of technological, intellectual, social, and emotional work sketched here mean that patients, their families, caregivers, and other health care providers will be faced with an increasing range of tasks in the domain of health care, which we have summarized in a list (Table 1). This list of tasks is not meant to be exhaustive but rather to make explicit some of the principal kinds of work involved in making data matter medically. Many of these concerns overlap; we expect that new forms of expertise will continue to emerge along with clinical and technological advances.

**Table 1:** Outline of various types of data work with examples.

Types of data work	Why is this work needed?	Examples of data work in practice; ongoing and possible in the future
Supporting digital data practices	Engagement with health data is increasingly taking place outside the clinic, and it can also create digital divides*;* traditional means of managing and evaluating data are increasingly not suited to meet the realities of the digital age; persistent difficulties in assessing accuracy and appropriateness of diverse, unvalidated forms of health data.	Patients research and consider the implications of data; health practitioners assist in navigation of data relationships; creation of guidelines for how to evaluate new digital technologies or assess internet sources; identification of how digital interaction can create new patterns of exclusion.
The work of interpretation and contextualization	Unclear what biometric data collected via devices such as wearables or smartphones will mean for medical practice; misleading or false health information is often shared on the internet; the algorithms that produce data are neither objective nor intrinsically fair; the full implications of diverse, unregulated health information are often difficult for users to discern or anticipate.	Expert guidance on how to decide which devices and resulting data are reliable and relevant for a given context; research on reliability of commercial devices; provision of prescreening and assistance to make digital health tools meaningful for individual patients; identification of biases built into algorithms of datasets, devices, and models.
The work of inclusion and interaction	Data are increasingly accessible, distributed, revealing, and reidentifiable, creating new ethical concerns; perceived benefits of the data-driven medicine and the social, economic, and health-related concerns vary by actor; patient experience of digital tools affects self-management of chronic conditions and well-being.	Support for patients in determining their priorities, needs, and wishes with regard to their digital health activities and data collection and use; facilitation of conversations between differently motivated parties about aims, goals, and interests.

Yet what is clear is that the problems accompanying these demands are currently underappreciated. This raises the question of who should be tasked with the increasing interpretation needs of data in the health care domain. Visions of data-rich medicine often imply that doctors should or will take on this work, as reflected in frequent calls for better genomic or data literacy for health care professionals. In the past decade, there have been numerous calls for more training in several of the domains mentioned above, such as ethical concerns surrounding the communication of genetic data and related health risks to patients [42], or counseling patients about the advantages and pitfalls of Web-based or commercial sources of health information [69]. Some, such as Celi et al, call for increased training of medical students and residents in order to “creat[e] a medical culture that is aware of and respectful of the importance and potential power of data for supporting and improving both practice and research may be the most important and ultimately effective element” [53]. At the moment, although health care professionals are seen as the first in line to take on this additional work, allowances are not made in schedules or training to accommodate meaningful engagement with the social complexities of data in medicine. Even if actors find the time to engage in the various types of data work, not all can acquire the necessary skills. Finally, many of the tasks described above take place outside health professionals’ sphere of influence entirely.

Throughout this paper, we have proposed a few possible ways of addressing the emerging forms of data work identified here, ranging from the creation of a new profession dedicated to help both patients and providers assess and understand diverse kinds of health data, to greater involvement and creation of guidelines by scientific and academic societies, to raising expectations through regulatory frameworks for how mechanisms such as informed consent are operationalized across novel research practices. However, none of these approaches alone will be sufficient for taking on the myriad aspects of data work that we have outlined, as well as those that will continue to emerge in the future. Although the focus of this paper has been on the identification of the contours of the phenomenon we are calling *data work*, further attention is needed to analyze and consider other solutions for addressing these concerns. Importantly, some aspects of data work can neither be delegated to professionals nor addressed completely through better guidelines or greater public discourse. Hence, the current landscape of big data in medicine remains open for new proposals, such as how such work can or should be acknowledged or even reimbursed. What other tools—conceptual, analytic, instructive, or collaborative—would be helpful for navigating increasingly complex data use? What would be a fair division of work? What responsibilities should corporations using health data have, beyond compliance with data protection regulations? Our intent is that by making these forms of work more explicit and transparent, more appropriate ways of addressing data work can be devised in future.

## Conclusions

In addition to the established challenges surrounding data collection, storage, analysis, and security, pressing questions have arisen around: how to enable the appropriate use of technologies and engagement with health data outside of the structured environment of health care; what the utility, quality, and possibilities of data collected from wearable devices or smartphones will be for clinical practice; strategies to avoid the digital health divide; how to distinguish data noise from clinically actionable health resources for patients; how to contextualize health data gained through Web-based tests or digital technologies; and how to foster conversations surrounding the ethical concerns of big data between different stakeholders in health care and society. Of course, the various forms of work included within the categories of supporting digital tool use, contextualization, and inclusion and integration cannot be neatly disentangled. Conversations between different actors in the health care domain are necessary to determine what types of data and data use are feasible, ethical, and cost-effective in particular situations. Although we expect that AI applications such as deep learning will be of great help in matters such as the interpretation of data, the analysis above has shown that the task of interpretation is not something that can be devolved to machines entirely.

A critical thread that runs throughout the forms of data work identified here is that of context: data work does not involve questions of absolutes but rather of contingencies. What is relevant, important, or significant for one individual may not apply to the next. Data, just like the experience of health and illness, are profoundly dependent upon the social world in which they exist. As we have shown in this paper, the turn toward data-rich health care has created new forms of data work and expertise. Data work needs to be named and recognized as the human endeavors that make digital advances meaningful in medicine. We argue that greater attention is needed for the very craft of deriving choices, narratives, and practices from our data and that the current medical system is not equipped to take on this challenge alone. If the great potential of data-rich medicine to improve future clinical care is to be realized, the new data work that patients, health professionals, and other actors increasingly contribute must be recognized as an important and multifaceted task.

## Abbreviations

AI: artificial intelligence
EU: European Union
GDPR: General Data Protection Regulation
HIC: health information counselor
HIPAA: Health Insurance Portability and Accountability Act

## References

[1] Neff G (2013). Why big data won't cure us. Big Data.

[2] Reddy MC, Gorman P, Bardram J (2011). Special issue on supporting collaboration in healthcare settings: the role of informatics. Int J Med Inform.

[3] Aarts J (2013). A sociotechnical perspective of health information technology. Int J Med Inform.

[4] Bjørn P, Kensing F (2013). Special issue on information infrastructures for healthcare: the global and local relation. Int J Med Inform.

[5] Leonelli S (2014). Data interpretation in the digital age. Perspect Sci.

[6] Stein R (2018). National Public Radio.

[7] Kolata G, Murphy H (2018). The New York Times.

[8] Saey TH (2019). Science News.

[9] Sifferlin A (2017). Time.

[10] O'Neil C (2016). Weapons of Math Destruction: How Big Data Increases Inequality and Threatens Democracy.

[11] (2017). Deutscher Ethikrat.

[12] (2014). Nuffield Council on Bioethics.

[13] Su P (2013). Direct-to-consumer genetic testing: a comprehensive view. Yale J Biol Med.

[14] Richter G, Krawczak M, Lieb W, Wolff L, Schreiber S, Buyx A (2018). Broad consent for health care–embedded biobanking: understanding and reasons to donate in a large patient sample. Genet Med.

[15] Whetton A (2019). Institute of Infectious Disease and Molecular Medicine.

[16] Neff G, Tanweer A, Fiore-Gartland B, Osburn L (2017). Critique and contribute: a practice-based framework for improving critical data studies and data science. Big Data.

[17] Leonelli S (2016). Data-Centric Biology: A Philosophical Study.

[18] Ebeling MF (2016). Healthcare and Big Data: Digital Specters and Phantom Objects.

[19] Tempini N, Leonelli S (2018). Genomics and big data in biomedicine. Routledge Handbook of Genomics, Health and Society.

[20] Strauss AL, Fagerhaugh S, Suczek B, Wiener C (1982). The work of hospitalized patients. Soc Sci Med.

[21] Strauss A (1988). The articulation of project work: an organizational process. Sociol Q.

[22] Strauss AL (1997). Social Organization of Medical Work.

[23] Clarke A, Mamo L, Fishman JR, Shim JK, Fosket JR (2003). Biomedicalization: technoscientific transformations of health, illness, and US biomedicine. Am Sociol Rev.

[24] Stacey M (1984). Who are the health workers? Patients and other unpaid workers in health care. Econ Ind Democr.

[25] Prainsack B (2017). Personalized Medicine: Empowered Patients in the 21st Century?.

[26] Knoppers BM, Thorogood AM (2017). Ethics and big data in health. Curr Opin Syst Biol.

[27] Belle A, Thiagarajan R, Soroushmehr SM, Navidi F, Beard DA, Najarian K (2015). Big data analytics in healthcare. Biomed Res Int.

[28] Loiselle CG, Ahmed S (2017). Is connected health contributing to a healthier population?. J Med Internet Res.

[29] Hesse BW, Greenberg AJ, Rutten LJ (2016). The role of internet resources in clinical oncology: promises and challenges. Nat Rev Clin Oncol.

[30] Ramirez V, Johnson E, Gonzalez C, Ramirez V, Rubino B, Rossetti G (2016). Assessing the use of mobile health technology by patients: an observational study in primary care clinics. JMIR Mhealth Uhealth.

[31] Klerings I, Weinhandl AS, Thaler KJ (2015). Information overload in healthcare: too much of a good thing?. Z Evid Fortbild Qual Gesundhwes.

[32] Kung J, Wu CT (2016). Leveling the playing field: closing the gap in public awareness of genetics between the well served and underserved. Hastings Cent Rep.

[33] Peng W, Kanthawala S, Yuan S, Hussain SA (2016). A qualitative study of user perceptions of mobile health apps. BMC Public Health.

[34] Del Savio L, Prainsack B, Buyx A (2017). Motivations of participants in the citizen science of microbiomics: data from the British gut project. Genet Med.

[35] Nguyen A, Mosadeghi S, Almario CV (2017). Persistent digital divide in access to and use of the internet as a resource for health information: results from a California population-based study. Int J Med Inform.

[36] Hong YA, Zhou Z, Fang Y, Shi L (2017). The digital divide and health disparities in China: evidence from a national survey and policy implications. J Med Internet Res.

[37] Lorence DP, Park H, Fox S (2006). Racial disparities in health information access: resilience of the digital divide. J Med Syst.

[38] Fox S, Purcell K (2010). Pew Research Center.

[39] Casper M, Moore LJ (2009). Missing Bodies: The Politics Of Visibility.

[40] (2019). NHS.

[41] Gorman D, Kashner TM (2018). Medical graduates, truthful and useful analytics with big data, and the art of persuasion. Acad Med.

[42] Badalato L, Kalokairinou L, Borry P (2017). Third party interpretation of raw genetic data: an ethical exploration. Eur J Hum Genet.

[43] Annes JP, Giovanni MA, Murray MF (2010). Risks of presymptomatic direct-to-consumer genetic testing. N Engl J Med.

[44] Fiske A, Buyx A, Prainsack B (2019). Health information counselors: a new profession for the age of big data. Acad Med.

[45] Granado-Font E, Flores-Mateo G, Sorlí-Aguilar M, Montaña-Carreras X, Ferre-Grau C, Barrera-Uriarte ML, Oriol-Colominas E, Rey-Reñones C, Caules I, Satué-Gracia EM, OBSBIT Study Group (2015). Effectiveness of a smartphone application and wearable device for weight loss in overweight or obese primary care patients: protocol for a randomised controlled trial. BMC Public Health.

[46] Lewis Jr GK, Langer MD, Henderson Jr CR, Ortiz R (2013). Design and evaluation of a wearable self-applied therapeutic ultrasound device for chronic myofascial pain. Ultrasound Med Biol.

[47] Mummah S, Robinson TN, Mathur M, Farzinkhou S, Sutton S, Gardner CD (2017). Effect of a mobile app intervention on vegetable consumption in overweight adults: a randomized controlled trial. Int J Behav Nutr Phys Act.

[48] Macdonald GG, Townsend AF, Adam P, Li LC, Kerr S, McDonald M, Backman CL (2018). eHealth technologies, multimorbidity, and the office visit: qualitative interview study on the perspectives of physicians and nurses. J Med Internet Res.

[49] Anstey Watkins J, Goudge J, Gómez-Olivé FX, Huxley C, Dodd K, Griffiths F (2018). mHealth text and voice communication for monitoring people with chronic diseases in low-resource settings: a realist review. BMJ Glob Health.

[50] Banbury A, Nancarrow S, Dart J, Gray L, Parkinson L (2018). Telehealth interventions delivering home-based support group videoconferencing: systematic review. J Med Internet Res.

[51] Turrini M, Prainsack B (2016). Beyond clinical utility: the multiple values of DTC genetics. Appl Transl Genom.

[52] Anderson K, Burford O, Emmerton L (2016). Mobile health apps to facilitate self-care: a qualitative study of user experiences. PLoS One.

[53] Celi LA, Davidzon G, Johnson AE, Komorowski M, Marshall DC, Nair SS, Phillips CT, Pollard TJ, Raffa JD, Salciccioli JD, Salgueiro FM, Stone DJ (2016). Bridging the health data divide. J Med Internet Res.

[54] Graber ML (2013). The incidence of diagnostic error in medicine. BMJ Qual Saf.

[55] Madathil KC, Rivera-Rodriguez AJ, Greenstein JS, Gramopadhye AK (2015). Healthcare information on YouTube: a systematic review. Health Informatics J.

[56] Murray E, Lo B, Pollack L, Donelan K, Catania J, White M, Zapert K, Turner R (2003). The impact of health information on the internet on the physician-patient relationship: patient perceptions. Arch Intern Med.

[57] Fernandez-Luque L, Karlsen R, Bonander J (2011). Review of extracting information from the social web for health personalization. J Med Internet Res.

[58] Syed-Abdul S, Fernandez-Luque L, Jian WS, Li YC, Crain S, Hsu MH, Wang YC, Khandregzen D, Chuluunbaatar E, Nguyen PA, Liou DM (2013). Misleading health-related information promoted through video-based social media: anorexia on YouTube. J Med Internet Res.

[59] Fiske A, Prainsack B, Buyx A (2019). Survey of Health Care Practitioner Assessments of Self Care in Schleswig-Holstein, Germany.

[60] Aitken M, Lyle J (2015). IQVIA.

[61] (2015). The European Commission.

[62] Pasquale F (2016). The Black Box Society: The Secret Algorithms That Control Money And Information.

[63] Becker S, Miron-Shatz T, Schumacher N, Krocza J, Diamantidis C, Albrecht UV (2014). mHealth 2.0: experiences, possibilities, and perspectives. JMIR Mhealth Uhealth.

[64] Harris A, Kelly S, Wyatt S (2016). CyberGenetics: Health Genetics and New Media.

[65] Floridi L, Cowls J, Beltrametti M, Chatila R, Chazerand P, Dignum V, Luetge C, Madelin R, Pagallo U, Rossi F, Schafer B, Valcke P, Vayena E (2018). AI4People-an ethical framework for a good AI society: opportunities, risks, principles, and recommendations. Minds Mach (Dordr).

[66] Fiske A, Henningsen P, Buyx A (2019). Your robot therapist will see you now: ethical implications of embodied artificial intelligence in psychiatry, psychology, and psychotherapy. J Med Internet Res.

[67] Ruckenstein M, Schüll ND (2017). The datafication of health. Annu Rev Anthropol.

[68] Kaye J, Curren L, Anderson N, Edwards K, Fullerton SM, Kanellopoulou N, Lund D, MacArthur DG, Mascalzoni D, Shepherd J, Taylor PL, Terry SF, Winter SF (2012). From patients to partners: participant-centric initiatives in biomedical research. Nat Rev Genet.

[69] (2010). Nuffield Council on Bioethics.

[70] Capper D, Jones DT, Sill M, Hovestadt V, Schrimpf D, Sturm D, Koelsche C, Sahm F, Chavez L, Reuss DE, Kratz A, Wefers AK, Huang K, Pajtler KW, Schweizer L, Stichel D, Olar A, Engel NW, Lindenberg K, Harter PN, Braczynski AK, Plate KH, Dohmen H, Garvalov BK, Coras R, Hölsken A, Hewer E, Bewerunge-Hudler M, Schick M, Fischer R, Beschorner R, Schittenhelm J, Staszewski O, Wani K, Varlet P, Pages M, Temming P, Lohmann D, Selt F, Witt H, Milde T, Witt O, Aronica E, Giangaspero F, Rushing E, Scheurlen W, Geisenberger C, Rodriguez FJ, Becker A, Preusser M, Haberler C, Bjerkvig R, Cryan J, Farrell M, Deckert M, Hench J, Frank S, Serrano J, Kannan K, Tsirigos A, Brück W, Hofer S, Brehmer S, Seiz-Rosenhagen M, Hänggi D, Hans V, Rozsnoki S, Hansford JR, Kohlhof P, Kristensen BW, Lechner M, Lopes B, Mawrin C, Ketter R, Kulozik A, Khatib Z, Heppner F, Koch A, Jouvet AA, Keohane C, Mühleisen H, Mueller W, Pohl U, Prinz M, Benner A, Zapatka M, Gottardo NG, Driever PH, Kramm CM, Müller HL, Rutkowski S, von Hoff K, Frühwald MC, Gnekow A, Fleischhack G, Tippelt S, Calaminus G, Monoranu CM, Perry A, Jones C, Jacques TS, Radlwimmer B, Gessi M, Pietsch T, Schramm J, Schackert G, Westphal M, Reifenberger G, Wesseling P, Weller M, Collins VP, Blümcke I, Bendszus M, Debus J, Huang A, Jabado N, Northcott PA, Paulus W, Gajjar A, Robinson GW, Taylor MD, Jaunmuktane Z, Ryzhova M, Platten M, Unterberg A, Wick W, Karajannis MA, Mittelbronn M, Acker T, Hartmann C, Aldape K, Schüller U, Buslei R, Lichter P, Kool M, Herold-Mende C, Ellison DW, Hasselblatt M, Snuderl M, Brandner S, Korshunov A, von Deimling A, Pfister SM (2018). DNA methylation-based classification of central nervous system tumours. Nature.

[71] Klingmüller U (2018). Ethics, bio-politics and regulation of precision medicine. https://tinyurl.com/yxh7thkv.

[72] Schueller SM, Washburn JJ, Price M (2016). Exploring mental health providers' interest in using web and mobile-based tools in their practices. Internet Interv.

[73] Beauchamp TL, Childress JF (2012). Principles Of Biomedical Ethics.

[74] Rumbold JM, Pierscionek B (2017). The effect of the general data protection regulation on medical research. J Med Internet Res.

[75] Thompson B (2016). Welcome Trust.

[76] Dreyer NA, Blackburn S, Hliva V, Mt-Isa S, Richardson J, Jamry-Dziurla A, Bourke A, Johnson R (2015). Balancing the interests of patient data protection and medication safety monitoring in a public-private partnership. JMIR Med Inform.

[77] (2015). US Food and Drug Administration.

[78] (2017). LegiTech.

[79] Snell E (2018). Health IT Security.

[80] McMahon A, Buyx A, Prainsack B (2019). Big data governance needs more collective responsibility: the role of harm mitigation in the governance of data use in medicine and beyond. Med Law Rev.

[81] Sanderson SC, Brothers KB, Mercaldo ND, Clayton EW, Antommaria AH, Aufox SA, Brilliant MH, Campos D, Carrell DS, Connolly J, Conway P, Fullerton SM, Garrison NA, Horowitz CR, Jarvik GP, Kaufman D, Kitchner TE, Li R, Ludman EJ, McCarty CA, McCormick JB, McManus VD, Myers MF, Scrol A, Williams JL, Shrubsole MJ, Schildcrout JS, Smith ME, Holm IA (2017). Public attitudes toward consent and data sharing in biobank research: a large multi-site experimental survey in the US. Am J Hum Genet.

[82] Kayyali R, Hesso I, Ejiko E, Gebara SN (2017). A qualitative study of telehealth patient information leaflets (TILs): are we giving patients enough information?. BMC Health Serv Res.

[83] Prainsack B, Buyx A (2017). Solidarity in Biomedicine and Beyond.

[84] Spencer K, Sanders C, Whitley EA, Lund D, Kaye J, Dixon WG (2016). Patient perspectives on sharing anonymized personal health data using a digital system for dynamic consent and research feedback: a qualitative study. J Med Internet Res.

[85] Essén A, Scandurra I, Gerrits R, Humphrey G, Johansen MA, Kierkegaard P, Koskinen J, Liaw ST, Odeh S, Ross P, Ancker JS (2017). Patient access to electronic health records: differences across ten countries. Health Policy Technol.

[86] Wass S, Vimarlund V, Ros A (2019). Exploring patients' perceptions of accessing electronic health records: innovation in healthcare. Health Informatics J.

[87] Pormeister K (2017). Genetic data and the research exemption: is the GDPR going too far?. Int Data Priv Law.

